# Enhancing cold tolerance in tobacco through endophytic symbiosis with *Piriformospora indica*

**DOI:** 10.3389/fpls.2024.1459882

**Published:** 2024-10-25

**Authors:** Han Li, Zhiyao Wang, Yongxu Yu, Weichang Gao, Jingwei Zhu, Heng Zhang, Xiang Li, Yanxia Liu

**Affiliations:** ^1^ Upland Flue-cured Tobacco Quality and Ecology Key Laboratory, Guizhou Academy of Tobacco Science, Guiyang, China; ^2^ College of Tobacco Science, Guizhou University, Guiyang, China; ^3^ Technology Research and Development Center, Zunyi Branch of Guizhou Tobacco Company, Zunyi, China; ^4^ Tobacco Leaf Administration Office, Guizhou Branch Company of China Tobacco Corporation, Guiyang, China

**Keywords:** *Piriformospora indica*, tobacco cold resistance, reactive oxygen species, osmolytes, photosynthesis, N assimilation

## Abstract

Tobacco, a warm-season crop originating from the Americas, is highly susceptible to cold stress. The utilization of symbiotic fungi as a means to bolster crops’ resilience against abiotic stresses has been proven to be a potent strategy. In this study, we investigated the effect of endophytic fungus *Piriformospora indica* on the cold resistance of tobacco. When exposed to cold stress, the colonization of *P.indica* in tobacco roots effectively stimulates the activity of superoxide dismutase (SOD), catalase (CAT), peroxidase (POD), and ascorbate peroxidase (APX). This, in turn, reduces the accumulation of reactive oxygen species (ROS), thereby mitigating oxidative damage. Additionally, *P. indica* elevates the levels of osmolytes, such as soluble sugars, proline, and soluble proteins, thus facilitating the restoration of osmotic balance. Under cold stress conditions, *P. indica* also induces the expression of cold-responsive genes. Furthermore, this fungus not only enhances photosynthesis in tobacco by stimulating the synthesis of photosynthetic pigments, strengthening Rubisco activity, and elevating PSII efficiency, but also fortifies tobacco’s nitrogen assimilation by inducing the expression of nitrate transporter gene and activating enzymes related to nitrogen assimilation. Consequently, this synergistic optimization of nitrogen and carbon assimilation provides a solid material and energetic foundation for tobacco plants to withstand cold stress. Our study demonstrates that a mycorrhizal association between *P. indica* and tobacco seedlings provides multifaceted protection to tobacco plants against low-temperature stress and offers a valuable insight into how *P. indica* enhances the cold tolerance of tobacco.

## 1 Introduction

Temperature is a core environmental factor that shapes the life cycle and yield of plants, and its variations have profound effects on plant growth (Gray and Brady, 2016). It is widely recognized that plant productivity experiences a significant and disproportionate decrease when exposed to cold stress (Pearce, 2001). In such stressful environments, plants tend to generate a heightened quantity of reactive oxygen species, which are capable of causing oxidative damage to cells (You and Chan, 2015; Karami-Moalem et al., 2018). Concurrently, low temperatures hinder the synthesis of chlorophyll, which in turn compromises photosynthetic efficiency (Hajihashemi et al., 2018; Li et al., 2024). Moreover, exposure to low temperatures results in plant dehydration, decreased membrane fluidity, and disturbances in intracellular calcium homeostasis (Conde et al., 2011; Shi et al., 2014; Nechaeva et al., 2017; Agurla et al., 2018; Yuan et al., 2018). Most importantly, cold stress disrupts enzymatic activities within plants, negatively impacting respiratory processes and material metabolism, ultimately affecting the overall health and yield of the plants (Thakur et al., 2010).

To adapt to low-temperature stress, plants employ various defensive mechanisms. When subjected to chilly environments, plants accumulate osmolytes such as proline, soluble sugars, and soluble proteins (Kontunen-Soppela et al., 2002; Hayat et al., 2012; Jogawat, 2019; Ghosh et al., 2021; Xu et al., 2022). These substances are crucial for maintaining normal osmotic pressure within cells, thus effectively preventing cell damage due to dehydration. Concurrently, plants boost the activity of antioxidant enzymes, such as SOD and POD, to eliminate excess ROS (Gill and Tuteja, 2010; Adhikari et al., 2022; Wei et al., 2022). This action mitigates the oxidative damage caused by cold stress to plant cells. Additionally, elevated calcium ion levels regulate protein phosphorylation, which in turn stimulates the expression of cold resistance-related genes and facilitates the synthesis of specific proteins, ultimately bolstering the plant’s tolerance to cold stress (Saijo et al., 2000; Ma et al., 2015; Yuan et al., 2018). It is noteworthy that the ICE-CBF-COR pathway mediates plant cold tolerance (Zhao et al., 2016; Shi et al., 2018; Liu et al., 2019). Under freezing conditions, this pathway swiftly activates *CBF* (C-repeat-binding factor) expression, and the encoded protein directly modulates a set of *COR* (cold-regulated) genes, significantly bolstering the plant’s frost resistance. The transport and metabolism of inorganic ions, carbohydrates, and lipids also undergo changes in response to cold stress, collectively forming an effective defense against low temperatures in plants (Hurry et al., 2000; Trischuk et al., 2014; Barrero-Sicilia et al., 2017; Adhikari et al., 2022).

Multiple studies have consistently demonstrated that symbiotic fungi exhibit a pronounced beneficial effect on plants’ cold resistance or tolerance. Arbuscular mycorrhizal fungi have been shown to play a vital role in augmenting the photosynthesis and antioxidant capabilities of plants such as cucumber, rice, and corn (Charest et al., 1993; Chen et al., 2013; Liu et al., 2013). This, in turn, endows these plants with the resilience to withstand low temperatures. Meanwhile, *Piriformospora indica*, a symbiotic fungus with remarkable growth-promoting potential, has garnered attention for its performance in aiding host plants to withstand abiotic stress (Gill et al., 2016; Aslam et al., 2019). Under low-temperature conditions, *P. indica* is able to elevate the antioxidant capacity of *Phaseolus vulgaris* and barley, thereby fostering their growth (Murphy et al., 2014; Alizadeh Frutan et al., 2016). Li et al. (2021) pointed out that *P. indica* can enhance the cold tolerance of bananas by stimulating the antioxidant capacity of leaves, facilitating soluble sugar accumulation, and activating the expression of cold-responsive genes. Furthermore, Jiang et al. (2020) also noted that *P. indica* can reinforce the frost resistance of *Arabidopsis thaliana* by upregulating the expression of genes in the CBF-dependent pathway, specifically *ICE1* and *CBF1*.

Tobacco, a warm-season crop originating from the Americas, not only serves as an important model plant in biological research but also stands as a significant economic crop. In high-latitude regions, tobacco seedlings are first cultivated in greenhouses and then transplanted to the field in early spring. However, due to tobacco’s sensitivity to ambient temperature, exposure to cold stress during seedling cultivation and transplantation can significantly reduce germination rates, retard growth rates, and lower survival rates. These adverse effects severely hinder the normal growth and development of tobacco. Therefore, enhancing tobacco’s tolerance to cold stress is crucial. This study reveals that *P. indica* can significantly improve tobacco’s tolerance to cold stress. We delve into the multifaceted effects of *P. indica* on tobacco under low-temperature, including its promotional effect on photosynthesis, regulatory role in antioxidant enzyme activity and the accumulation of osmolytes, enhancement of nitrogen absorption and assimilation, and influence on the expression of cold-responsive genes. The findings of this study not only provide new insights into how endophytic fungi regulate plant cold resistance under low-temperature but also have important practical implications for applying these beneficial fungi as inoculants to enhance the cold resistance of crops.

## 2 Materials and methods

### 2.1 Plant materials and inoculation of *P. indica*

The inoculation of tobacco with *P. indica* was conducted based on the methodology outlined by Li et al. (2022). The mycelium of *P. indica*, which had been cultivated in Potato Dextrose Broth for a duration of four days, was harvested using high-speed centrifugation at 10,000 rpm. The harvested mycelium was then resuspended in ddH_2_O, and the fungal pellet suspension was adjusted to an OD_600_ value of 0.1. Subsequently, this suspension was employed to irrigate uniformly grown, two-month-old tobacco seedlings. Meanwhile, the control seedlings underwent irrigation with an equivalent volume of ddH_2_O. Fifteen days post-inoculation, the tobacco roots were stained with 0.05% trypan blue under an optical microscope, according to the method described by Li et al. (2022). The establishment of a symbiotic relationship was ascertained through the observation of stained chlamydospores in the tobacco roots using a 10x objective lens.

### 2.2 Cold stress treatment of plant materials

Both inoculated and uninoculated tobacco plants with *P. indica* were cultivated in incubators maintained at temperatures of 25°C and 4°C, respectively. Both incubators were kept at a relative humidity of 60-80% and followed a photoperiod of 16-hour light/8-hour dark. Each treatment group consisted of six replicates. After two days of incubation, physiological and biochemical parameters, enzyme activity, as well as photosynthesis and chlorophyll fluorescence parameters were measured in roots and leaves. Additionally, RNA was extracted from samples for subsequent qRT-PCR analysis.

### 2.3 Determination of the physiological and biochemical parameters

Samples were ground into powder and used for the determination of physiological and biochemical parameters and enzyme activity. Commercial assay kits (Comin Biotechnology Co., Ltd., China) were utilized to determine the levels of superoxide anion (O_2_·^-^), H_2_O_2_, malondialdehyde, starch, soluble protein, soluble sugar, proline, relative electrical conductivity, as well as the activities of Rubisco, nitrate reductase (NR), nitrite reductase (NiR), glutamine synthetase (GS), NADH-glutamate oxaloacetate transaminase (NADH-GOGAT), superoxide dismutase (SOD), catalase (CAT), ascorbate peroxidase (APX), and peroxidase (POD). Chlorophyll and carotenoid content were assayed spectrophotometrically, referring to a previously established protocol by Lichtenthaler (1987). Additionally, Total nitrogen content was determined using the micro-Kjeldahl method after digesting the samples in H_2_SO_4_-H_2_O_2_. The obtained data were derived from four biological replicates.

### 2.4 Detection of key photosynthetic and chlorophyll fluorescence parameters

The photosynthetic and chlorophyll fluorescence parameters of the samples were measured using a LI-6400 portable photosynthesis system (LI-COR, Lincoln, Nebraska, USA). The photosynthetic parameters encompassed net photosynthetic rate (Pn), stomatal conductance (Gs), intercellular carbon dioxide concentration (Ci), and transpiration rate (Tr). Prior to the measurement of photosynthetic parameters, tobacco leaves were illuminated with the instrument’s built-in light source for 30 minutes at a photosynthetic photon flux density of 1000 μmolm^−2^·s^−1^. Chlorophyll fluorescence parameters consisted of the maximum photochemical efficiency of photosystem II (Fv/Fm), actual photochemical quantum efficiency of photosystem II (ΦPSII), photosynthetic electron transport rate (ETR), photochemical quenching coefficient (qP), and non-photochemical quenching coefficient (NPQ). Before measuring chlorophyll fluorescence parameters, the potted tobacco seedlings were kept in darkness for 40 minutes.

### 2.5 RNA extraction and qRT-PCR analysis

RNA was extracted from samples using the RNAprep Pure Plant Plus Kit (TIANGEN BIOTECH (BEIJING) CO., LTD). Subsequently, 1 μg of RNA was utilized for the synthesis of the first strand complementary DNA (cDNA) (PrimeScript™ RT reagent Kit with gDNA Eraser, Takara). Quantitative real-time polymerase chain reaction (qRT-PCR) was performed using the method described previously, employing the fluorescent quantitative PCR kit (TB Green® Premix Ex Taq™ II (Tli RNaseH Plus), Bulk) on the Applied Biosystems ViiA™ 7 real-time PCR instrument. The housekeeping gene *NtACTIN* served as an internal reference gene. The primers for qRT-PCR are listed in **Supplementary Table S1**.

### 2.6 Statistical analysis of data

Data were analyzed using Tukey’s test (p < 0.05) in GraphPad Prism version 4.

## 3 Results

### 3.1 *P. indica* enhances the tolerance of tobacco to cold stress

**Figure 1:**
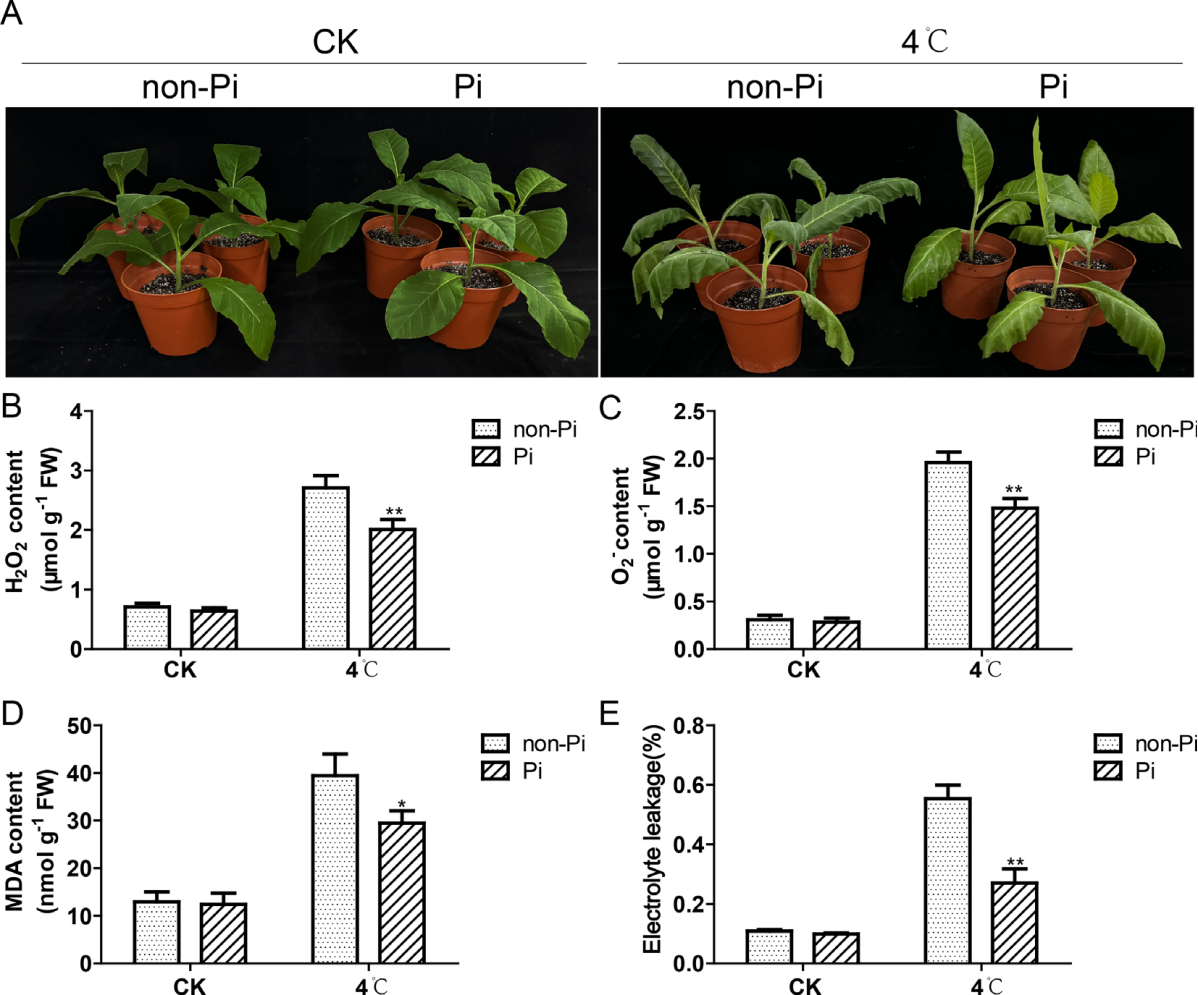
Effect of *P. indica* on phenotypes of tobacco leaves and its ability to mitigate oxidative stress under cold stress. **(A)** Effect of *P. indica* on phenotypes of tobacco leaves under cold stress; **(B)** H_2_O_2_ content in tobacco leaves; **(C)** O^-2^ content in tobacco leaves; **(D)** MDA content in tobacco leaves; **(E)** The relative electrolyte leakage rate of tobacco leaves; CK, control group; 4°C, treatment at 4°C; Pi, *P. indica*-colonized tobacco plants; non-Pi, control plants. Data are means (± SD), n=3. Significant differences between means were determined using Student’s t-test: *P<0.05, **P<0.01.

The *P. indica*-colonized tobacco and the uncolonized tobacco plants (control group) were placed in a 4°C incubator with a photoperiod of 16-hour light/8-hour dark for a two-day low-temperature treatment. As shown in **Figure 1A**, after cold stress treatment, there is a significant phenotype difference between the *P. indica*-inoculated tobacco and the control group. The control group showed obvious wilting symptoms and water-soaked spots, while the *P. indica*-inoculated tobacco exhibited much slighter injury symptoms. To gain a deeper understanding of the physiological disparities, we conducted further measurements of ROS and MDA levels in the leaves of both groups. The findings revealed that the control plants exhibited significantly higher levels of hydrogen peroxide and superoxide anion compared to the *P. indica*-inoculated tobacco (**Figures 1B, C**). Concurrently, the MDA content in the leaves of the control plants was also significantly higher than that of the *P. indica*-colonized tobacco, indicating that the control group suffered more severe oxidative damage under cold stress (**Figure 1D**). In addition, we also found that the electrolyte leakage rate in the control group was notably elevated compared to the *P. indica*-colonized tobacco, implying enhanced cell membrane stability in the *P. indica*-colonized plants under cold stress (**Figure 1E**).

### 3.2 Effect of *P. indica* on tobacco photosynthesis under cold stress

**Figure 2:**
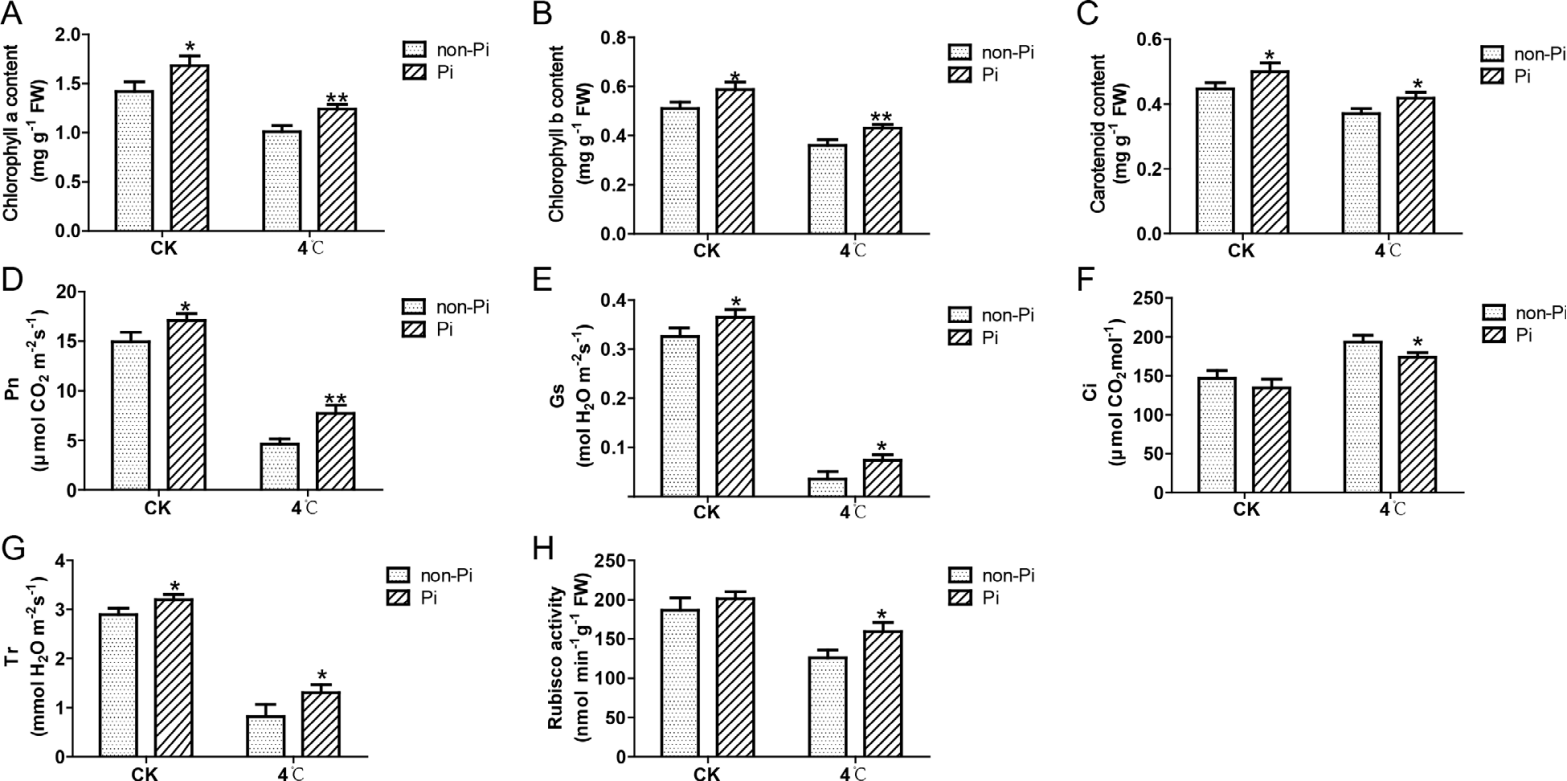
Effects of *P. indica* on photosynthesis in tobacco leaves under cold stress. **(A)** Chlorophyll a content in tobacco leaves; **(B)** Chlorophyll b content in tobacco leaves; **(C)** Carotenoid content in tobacco leaves; **(D)** Net photosynthetic rate (Pn) of tobacco leaves; **(E)** Stomatal conductance (Gs) of tobacco leaves; **(F)** Intercellular CO2 concentration (Ci) of tobacco leaves; **(G)** Transpiration rate (Tr) of tobacco leaves; **(H)** Rubisco activity in tobacco leaves; CK, control group; 4°C, treatment at 4°C; Pi, *P. indica*-colonized tobacco plants; non-Pi, control plants. Data are means (± SD), n=3. Significant differences between means were determined using Student’s t-test: *P<0.05, **P<0.01.

Cold stress is typically accompanied by detrimental impacts on photosynthesis. In this study, we examined photosynthetic pigments, net photosynthetic rate, stomatal conductance, intercellular carbon dioxide concentration, transpiration rate, and Rubisco activity in tobacco leaves under a 4°C environment. The results indicated that cold stress reduced the accumulation of chlorophyll a, chlorophyll b, and carotenoids in leaves of control plants, while inoculation with *P. indica* mitigated the loss of chlorophyll a, chlorophyll b, and carotenoids (**Figures 2A-C**). In comparison to the control group, tobacco inoculated with *P. indica* demonstrated elevated Pn, Gs, and Tr during cold stress, accompanied by a comparatively lower Ci (**Figures 2D-G**). Furthermore, the activity of the Rubisco enzyme was also significantly increased in tobacco plants colonized by *P. indica* (**Figure 2H**). These findings suggest that *P. indica* effectively enhances the photosynthetic capacity of tobacco under cold stress conditions.

**Figure 3:**
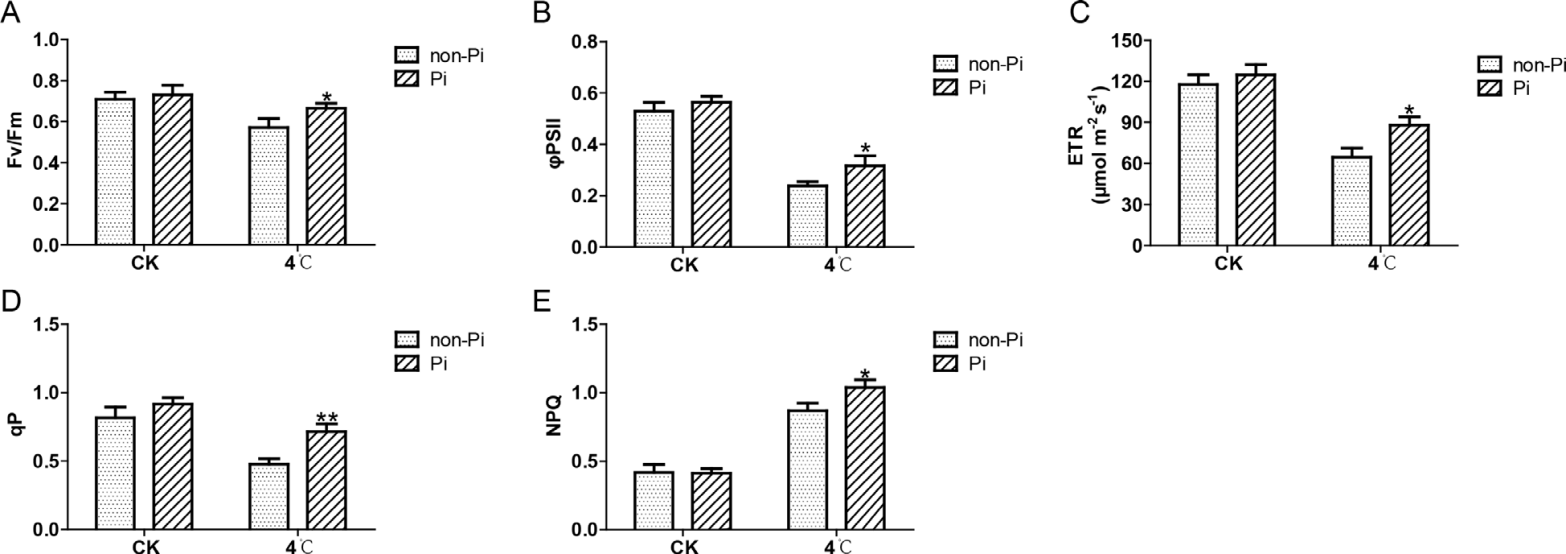
Effects of *P. indica* on fluorescence parameters in tobacco leaves under low cold stress. **(A)** Maximum photochemical efficiency of photosystem II (Fv/Fm); **(B)** Actual photochemical quantum efficiency of photosystem II (φPSII); **(C)** Photosynthetic electron transport rate (ETR); **(D)** Photochemical quenching coefficient (qP); **(E)** Non-photochemical quenching coefficient (NPQ); CK, control group; 4°C, treatment at 4°C; Pi, *P. indica*-colonized tobacco plants; non-Pi: control plants. Data are means (± SD), n=3. Significant differences between means were determined using Student’s t-test: *P<0.05, **P<0.01.

To further evaluate the positive role of *P. indica* in maintaining photosynthesis under cold stress, we measured the photosynthetic fluorescence parameters of tobacco leaves under cold stress, including Fv/Fm, ΦPSII, ETR, qP, and NPQ. As illustrated in **Figure 3**, after cold stress treatment, the Fv/Fm, Φ PSII, ETR, qP, and NPQ values of the control plants were significantly lower than those of *P. indica*-colonized tobacco (**Figures 3A-E**). These findings indicate that *P. indica* can effectively enhance the efficiency of the photosystem II in tobacco leaves under cold stress.

### 3.3 Effects of *P. indica* on the accumulation of osmolytes and the activity of antioxidant enzymes in tobacco under cold stress

**Figure 4:**
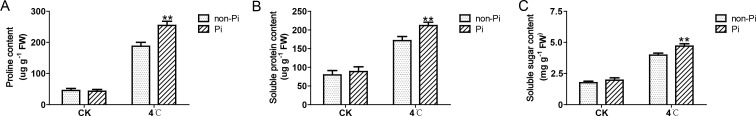
Effects of *P. indica* on the content of osmolytes in tobacco leaves under cold stress. **(A)** Proline content in tobacco leaves; **(B)** Soluble protein content in tobacco leaves; **(C)** Soluble sugar content in tobacco leaves; CK, control group; 4°C, treatment at 4°C; Pi, *P. indica*-colonized tobacco plants; non-Pi, control plants. Data are means (± SD), n=3. Significant differences between means were determined using Student’s t-test: *P<0.05, **P<0.01.

**Figure 5:**
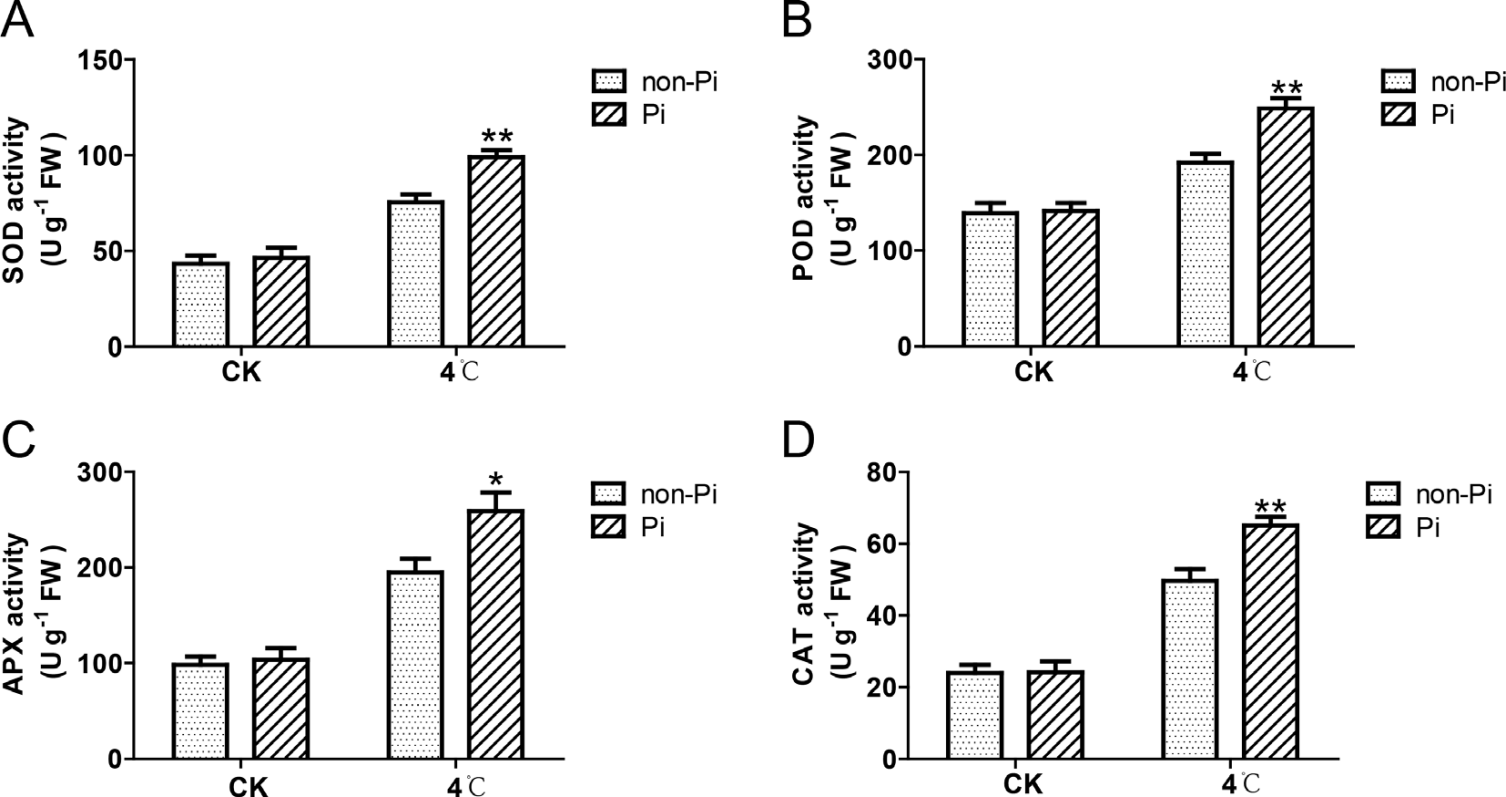
Effects of *P. indica* on the activity of antioxidant enzymes in tobacco leaves under cold stress. **(A)** Superoxide dismutase (SOD) activity in tobacco leaves; **(B)** Peroxidase (POD) activity in tobacco leaves; **(C)** Ascorbate peroxidase (APX) activity in tobacco leaves; **(D)** Catalase (CAT) activity in tobacco leaves; CK, control group; 4°C, treatment at 4°C; Pi, *P. indica*-colonized tobacco plants; non-Pi, control plants. Data are means (± SD), n=3. Significant differences between means were determined using Student’s t-test: *P<0.05, **P<0.01.

Plants adapt to cold stress by accumulating osmolytes such as soluble sugars, soluble proteins, and proline. In this study, we observed that the content of soluble sugars, soluble proteins, and proline in leaves of *P. indica*-colonized tobacco was significantly higher than that in the control group after cold stress treatment (**Figures 4A, C**). Plants can enhance the activity of antioxidant enzymes to eliminate excess ROS, thereby mitigating oxidative damage to plant cells. As illustrated in **Figure 5**, the activities of SOD, POD, CAT, and APX in tobacco leaves colonized by *P. indica* exhibited a significant increase compared to the control group following exposure to cold stress. These findings suggest that *P. indica* possesses the capability to stimulate the accumulation of osmolytes and enhance the activity of antioxidant enzymes in tobacco leaves under cold stress.

### 3.4 Effects of *P. indica* on nitrogen absorption and assimilation in tobacco under cold stress

**Figure 6:**
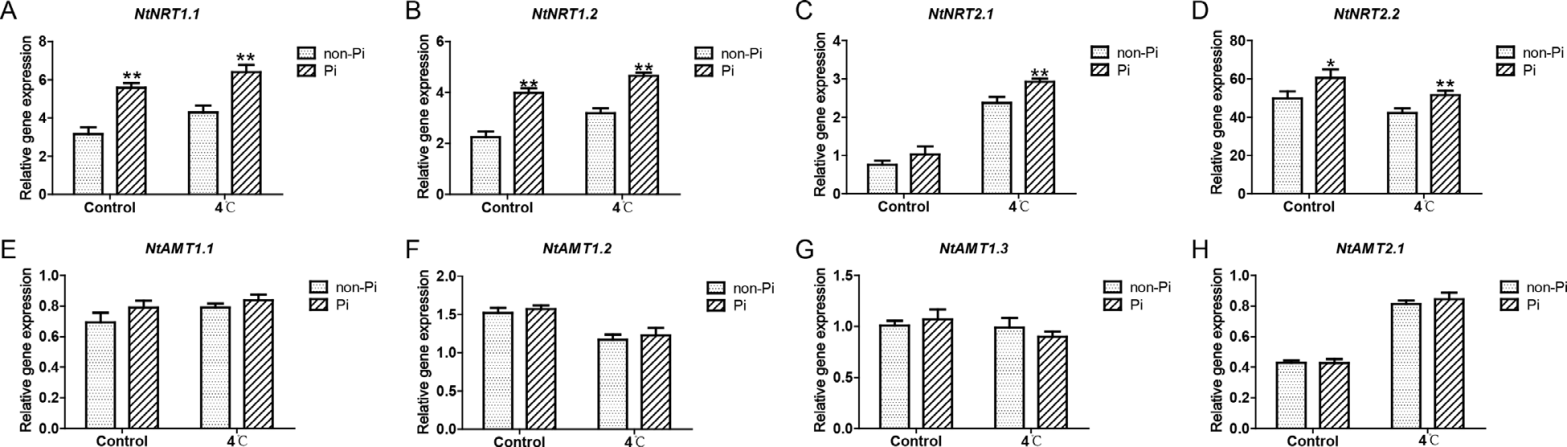
Effects of *P. indica* on the expression of ammonium and nitrate transporter genes in tobacco roots under cold stress. **(A-D)** The expression of nitrate transporter genes in tobacco roots; **(E-H)** The expression of ammonium transporter genes in tobacco roots; CK, control group; 4°C, treatment at 4°C; Pi, *P. indica*-colonized tobacco plants; non-Pi, control plants. Data are means (± SD), n=3. Significant differences between means were determined using Student’s t-test: *P<0.05, **P<0.01.

**Figure 7:**
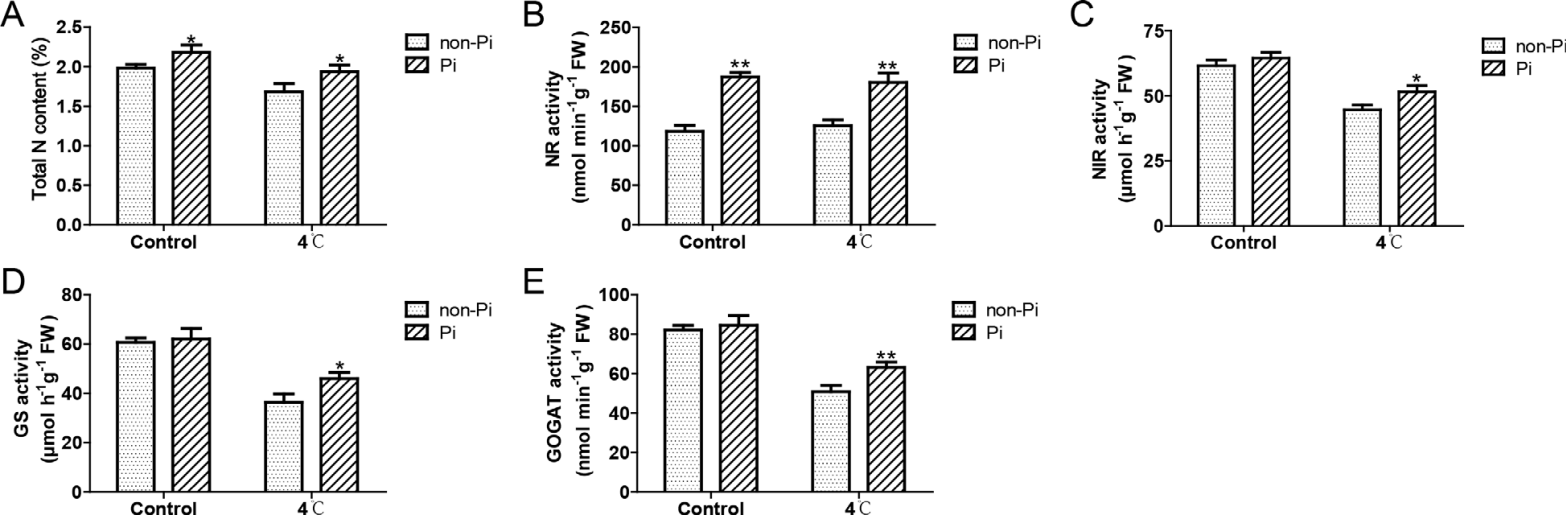
Effects of *P. indica* on the activity of key enzymes involved in nitrogen assimilation in tobacco under cold stress. **(A)** Total nitrogen content in tobacco roots; **(B)** Nitrate reductase (NR) activity in tobacco roots; **(C)** Nitrite reductase (NIR) activity in tobacco roots; **(D)** Glutamine synthetase (GS) activity in tobacco roots; **(E)** Glutamate synthase (GOGAT) activity in tobacco roots; CK, control group; 4°C, treatment at 4°C; Pi, *P. indica*-colonized tobacco plants; non-Pi, control plants. Data are means (± SD), n=3. Significant differences between means were determined using Student’s t-test: *P<0.05, **P<0.01.

It is well known that *P. indica* has the ability to enhance plants’ efficiency in absorbing external nutrients (Franken, 2012). In this study, we found that under normal temperature, colonization by *P. indica* can induce the expression of nitrate transporter genes (*NtNRT1.1*, *NtNRT1.2*, *NtNRT2.2*), and enhance the activity of nitrate reductase (NR), thereby increasing nitrogen content in the roots (**Figures 6A–H**; **Figures 7A, B**). Under cold stress conditions, tobacco plants colonized by *P. indica* exhibited significantly higher expression of nitrate transporter genes, including *NtNRT1.1*, *NtNRT1.2*, *NtNRT2.1*, and *NtNRT2.2*, compared to control plants (**Figures 6A–H**). Furthermore, the activities of nitrate reductase, nitrite reductase (NIR), glutamine synthetase (GS), and glutamate synthetase (GOGAT) were notably higher in the *P. indica*-colonized plants (**Figures 7B-E**). More importantly, the nitrogen content in the roots of these colonized plants was also significantly elevated compared to the control group (**Figure 7A**). These findings suggest that *P. indica* can enhance nitrogen absorption and assimilation processes in tobacco, even under cold stress.

### 3.5 Effects of *P. indica* on the expression of cold-responsive genes in tobacco

**Figure 8:**
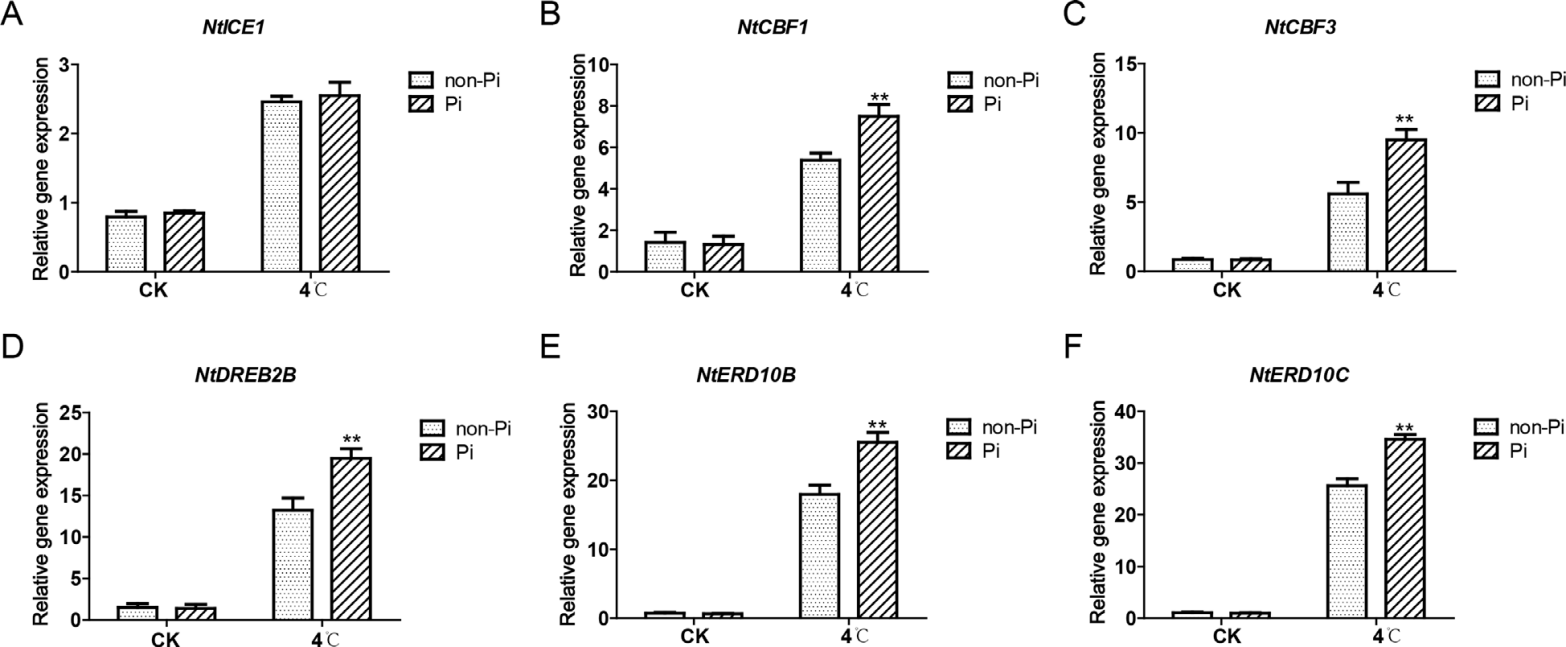
Effects of *P. indica* on the expression of cold-responsive genes in tobacco leaves under cold stress. **(A–F)** represent the qRT-PCR analysis results of NtICE1, NtCBF1, NtCBF3, NtDREB2B, NtERD10B, and NtERD10C in control plants and tobacco plants colonized by P. indica, respectively. CK, control group; 4°C, treatment at 4°C; Pi, *P. indica*-colonized tobacco plants; non-Pi, control plants. Data are means (± SD), n=3. Significant differences between means were determined using Student’s t-test: *P<0.05, **P<0.01.

The ICE-CBF-COR pathway plays a pivotal role in plant cold stress tolerance. Therefore, we investigated whether *P. indica* affects the expression of genes in the ICE-CBF-COR pathway in tobacco under cold stress. After low-temperature treatment, apart from *ICE1*, which showed no significant difference in expression between *P. indica*-colonized and non-colonized plants, the genes *NtCBF1*, *NtCBF3*, *NtDREB2B*, *NtERD10B*, and *NtERD10C* exhibited higher expression in *P. indica*-colonized tobacco (**Figures 8B-F**). This suggests that the cold stress tolerance induced by *P. indica* may be associated with the upregulation of *CBF* and *COR* expression.

## 4 Discussion

Cold stress, a significant factor limiting plant growth and yield, has profound negative impacts on up to 15% of agricultural land worldwide, leading to plant cell membrane rupture, loss of turgor, metabolic disorders, and even cell death (Thakur et al., 2010; Chaudhry and Sidhu, 2022). Recent extensive studies have revealed that cold stress disrupts the cellular homeostasis, disturbing the balance between the generation and scavenging of ROS, such as O_2_^-^ and H_2_O_2_, leading to an excessive buildup of intracellular ROS (You and Chan, 2015; Karami-Moalem et al., 2018). Such ROS surge can cause oxidative damage to lipids, proteins, and nucleic acids in plant tissues, further exacerbating the damage caused by cold stress to plants (Pennycooke et al., 2005). The antioxidant enzyme system is considered a crucial defense mechanism for plants to scavenge ROS under environmental stress (Navrot et al., 2007; Rajput et al., 2021). Among these enzymes, SOD specifically converts superoxide radicals into hydrogen peroxide and oxygen. Subsequently, hydrogen peroxide is effectively scavenged under the activity of APX, CAT, and POD (Gill and Tuteja, 2010). Current studies suggest that colonization by symbiotic fungi can enhance the antioxidant enzyme system of host plants, effectively maintaining ROS homeostasis, and thus mitigating the inhibitory effects caused by elevated ROS levels that accumulate during plant cellular processes, growth, and survival under stressful environment. For instance, AMF upregulates the activities of multiple antioxidant enzymes, including SOD, POD, CAT, and APX, and reduces the concentration of MDA, providing enhanced stability to the plasma membrane (Latef and Chaoxing, 2011; Huang et al., 2014; Li et al., 2019; He et al., 2020). In this study, we observed that cold stress triggers an outburst of ROS in tobacco leaves, leading to the generation of MDA and an increase in electrolyte leakage. However, inoculation with *P. indica* significantly enhances the activities of SOD, POD, CAT, and APX, thereby reducing MDA accumulation and alleviating electrolyte leakage. The lower MDA level and electrolyte leakage rate in these plants confirm that colonized plants suffer less from the cold stress. Similarly, in banana, colonization by *P. indica* significantly stimulates the activities of SOD, CAT, APX, and glutathione reductase (GR), effectively reducing the excessive accumulation of ROS under cold stress (Li et al., 2021). These results indicated that colonization by *P. indica* can enhance the antioxidant enzyme activities of host plants, thereby maintaining cell membrane stability under cold stress.

Cold stress often exerts detrimental effects through osmotic stress, and such damaging effects can be counteracted by the accumulation of osmolytes (such as soluble sugars, proline, and soluble proteins), thereby restoring osmotic balance (Kontunen-Soppela et al., 2002; Hayat et al., 2012; Jogawat, 2019; Ghosh et al., 2021; Xu et al., 2022). Furthermore, these osmolytes can preserve the integrity of cell membranes by directly participating in the scavenging of ROS, as well as by lowering the freezing point of plants, enabling them to better adapt to cold stress (Kontunen-Soppela et al., 2002; Hayat et al., 2012; Jogawat, 2019; Ghosh et al., 2021; Xu et al., 2022). This, in turn, enhances the oxidative tolerance of plants. Among them, proline enhances the stress tolerance of plants by maintaining osmotic balance, cell turgor, and indirectly regulating the metabolism of ROS (Hayat et al., 2012). Soluble sugars serve as an energy source, providing a basis for cellular metabolism, thereby enhancing the cold resistance of plants (Xu et al., 2022). Soluble proteins can bind with water to maintain cellular water content, thus reducing physiological water loss under cold stress (Kontunen-Soppela et al., 2002). In this study, the endophytic fungus *P. indica* significantly increased the contents of proline, soluble sugars, and soluble proteins in tobacco under low-temperature stress. This phenomenon has also been observed in *Arabidopsis thaliana* and banana, where *P. indica* enhances the contents of soluble proteins and proline in *Arabidopsis thaliana* (Jiang et al., 2020), and similarly, increases the levels of soluble sugars and proline in banana (Li et al., 2021). These findings indicate that *P. indica* stimulates the accumulation of osmolytes, thus improving the resistance of host plants to cold stress.

Chlorophyll plays a crucial role in capturing light energy during photosynthesis. While, cold stress significantly hinders the synthesis of chlorophyll or even causes its degradation, adversely affecting the photosynthesis of plants (Hajihashemi et al., 2018; Li et al., 2024). In this study, we found that inoculation with *P. indica* can effectively alleviate the decrease in chlorophyll and carotenoid content in tobacco under cold stress. This suggests that *P. indica* stimulates the synthesis of photosynthetic pigments, thereby reducing the negative impact of cold stress on plants. Furthermore, the results of this study indicate that photosynthesis in tobacco is severely inhibited under cold stress, which is particularly evident in the notable decreases observed in Pn, Gs, and Tr values. The reduction in Pn is likely associated with decreased Gs and the hindrance of CO_2_ entry into the leaves. However, colonization with *P. indica* enhances the Pn, Tr, and Gs of tobacco leaves, and simultaneously reduces the Ci. The decreased Ci value in tobacco inoculated with *P. indica* may be attributed to the fact that CO_2_ is an essential substrate for photosynthesis. The reduction in intercellular CO_2_ actually reflects a higher consumption of CO_2_ during the photosynthetic process, thereby promoting an increase in the Pn. In addition, cold stress can damage the enzymatic system involved in photosynthesis. Rubisco, a key rate-limiting enzyme in carbon assimilation during photosynthesis, experiences a significant decrease in activity under cold stress, thereby negatively affecting the process of photosynthesis (Salesse-Smith et al., 2020). However, our study found that inoculation with *P. indica* can increase the activity of Rubisco in tobacco, which is conducive to CO_2_ assimilation under cold stress.

Cold stress can also cause damage to the PSII reaction center, resulting in reduced photosynthetic activity, decreased rate of electron transfer in leaves, and increased photoinhibition (Wei et al., 2022). The rapid accumulation of ROS can further inhibit the synthesis of proteins necessary for PSII photodamage repair (such as D1 protein), exacerbating damage to the PSII reaction center (He and Chow, 2003). Fluorescence is an important indicator to measure PSII function and light capture efficiency. Under salt stress, inoculation with *P. indica* significantly enhances the PSII photochemical efficiency of tomatoes, manifested by notable increases in photosynthetic fluorescence parameters such as NPQ, qP, Fv/Fm, and ΦPSII (Ghorbani et al., 2018). Similarly, when plants are exposed to cadmium pollution, drought, or low temperatures, inoculation with *P. indica* can effectively enhance the PSII photochemical efficiency of the host plant, helping it better cope with various environmental pressures (Shahabivand et al., 2017; Li et al., 2021; Boorboori and Zhang, 2022). In this study, the tobacco colonized with *P. indica* exhibited less declines in Fv/Fm, ΦPSII, and ETR under cold stress compared to the uninoculated control group. The Fv/Fm ratio, which is sensitive to environmental stress, represents the maximum photochemical efficiency of PSII. Meanwhile, ΦPSII is closely related to the efficiency of light energy utilization in plants; when photosynthetically active radiation (PAR) is limited, a decrease in ΦPSII is indicative of a reduction in photosynthetic efficiency. ETR is indicative of the rate and efficiency of plants utilizing available light energy in the environment for photosynthesis. When illumination is insufficient, a decrease in ETR suggests a slowdown in the rate of photosynthesis. Additionally, qP, which represents the proportion of open PSII reaction centers, is positively correlated with plant photosynthetic activity. Low temperatures typically cause a decrease in qP (Banerjee and Roychoudhury, 2019). On the other hand, NPQ represents the photoprotection ability of plants to dissipate excess light energy through heat dissipation. The results show that after low-temperature treatment, the qP and NPQ values of tobacco leaves inoculated with *P. indica* were higher than those of the uninoculated control group. Considering *P. indica*’s ability to enhance the activity of antioxidant enzymes in tobacco, we hypothesize that it can alleviate the damage caused by excessive ROS generation to PSII reaction centers by activating the antioxidant enzyme defense system. Based on a comprehensive analysis of photosynthesis parameters, Rubisco activity, and fluorescence parameters, we believe that tobacco colonized by *P. indica* exhibits better photosynthetic capacity than uninoculated plants.

*P. indica* has been reported to enhance plant nitrogen use efficiency (Ansari et al., 2013). In this study, we observed that under cold stress, the colonization of tobacco plants by *P. indica* can significantly induce the expression of nitrate transporter genes, and enhance the activities of NR, NIR, GS, and GOGAT. The combined effect of these mechanisms promoted nitrogen uptake and assimilation in tobacco, resulting in increased nitrogen content in the roots. Nitrogen metabolism is a complex pathway that affects nearly all growth-determining processes in plants. Recent research has demonstrated that an increase in total nitrogen content and nitrogen assimilation plays a crucial role in enhancing plant tolerance to cold stress (Soualiou et al., 2023). This augmented nitrogen content promotes photosynthetic activity by enhancing nitrogen partitioning in the photosynthetic apparatus and inducing positive feedback effects on carbohydrate metabolism through nitrogen allocation to sink organs for growth (Yi et al., 2014; Bloom et al., 2010; Soualiou et al., 2023). In contrast, a reduction in nitrogen assimilation in plants directly impacts the nitrogen supply to the photosynthetic apparatus, thereby restricting the flow of nitrogen to the photosynthetic system (Yi et al., 2014; Bloom et al., 2010; Soualiou et al., 2023). This limitation not only undermines photosynthetic capacity but also significantly lowers the electron transport rate at the PSII reaction centers, posing challenges to plant growth and adaptability (Soualiou et al., 2023). Therefore, the enhancing effect of *P. indica* on nitrogen utilization efficiency in tobacco may be beneficial to the photosynthesis, so as to improve the assimilation of nitrogen and carbon in tobacco under cold stress, thus providing a material and energy foundation for tobacco to resist cold stress.

The cascade of cold signaling composed of ICE-CBF-COR plays a crucial role in plant cold resistance (Zhao et al., 2016; Shi et al., 2018; Liu et al., 2019). The CBF transcription factors, as downstream regulatory genes of *ICE1* (INDUCER OF CBF EXPRESSION 1), can be transiently and rapidly induced by cold stress. The induced CBFs can bind to the cis-acting elements of the promoters of *COR* (cold-regulated) genes, thereby activating the expression of *COR*s to enhance the plant’s ability to resist cold stress. This study demonstrates that, under cold stress, apart from *ICE1*, the expressions of *CBF1*, *CBF3*, and *DREB2B* (a homologous gene of *CBF1*) are significantly higher in tobacco colonized by *P. indica* than in the control group. The dehydration-responsive genes *NtERD10B* and *NtERD10C* are downstream regulatory factors of *CBF1* in tobacco, encoding LEA proteins (Shukla et al., 2006). Under cold stress, the expressions of *NtERD10B* and *NtERD10C* are also significantly higher in tobacco colonized by *P. indica* compared to the control group. Similarly, recent studies have shown that *P. indica* can enhance the tolerance of host plants to cold stress by activating the expression of *CBF*-dependent pathway genes (Jiang et al., 2020; Li et al., 2021). Therefore, we suggest that colonization by *P. indica* can enhance the expression of genes related to cold stress, thereby improving tobacco’s adaptability to cold stress.

**Figure 9:**
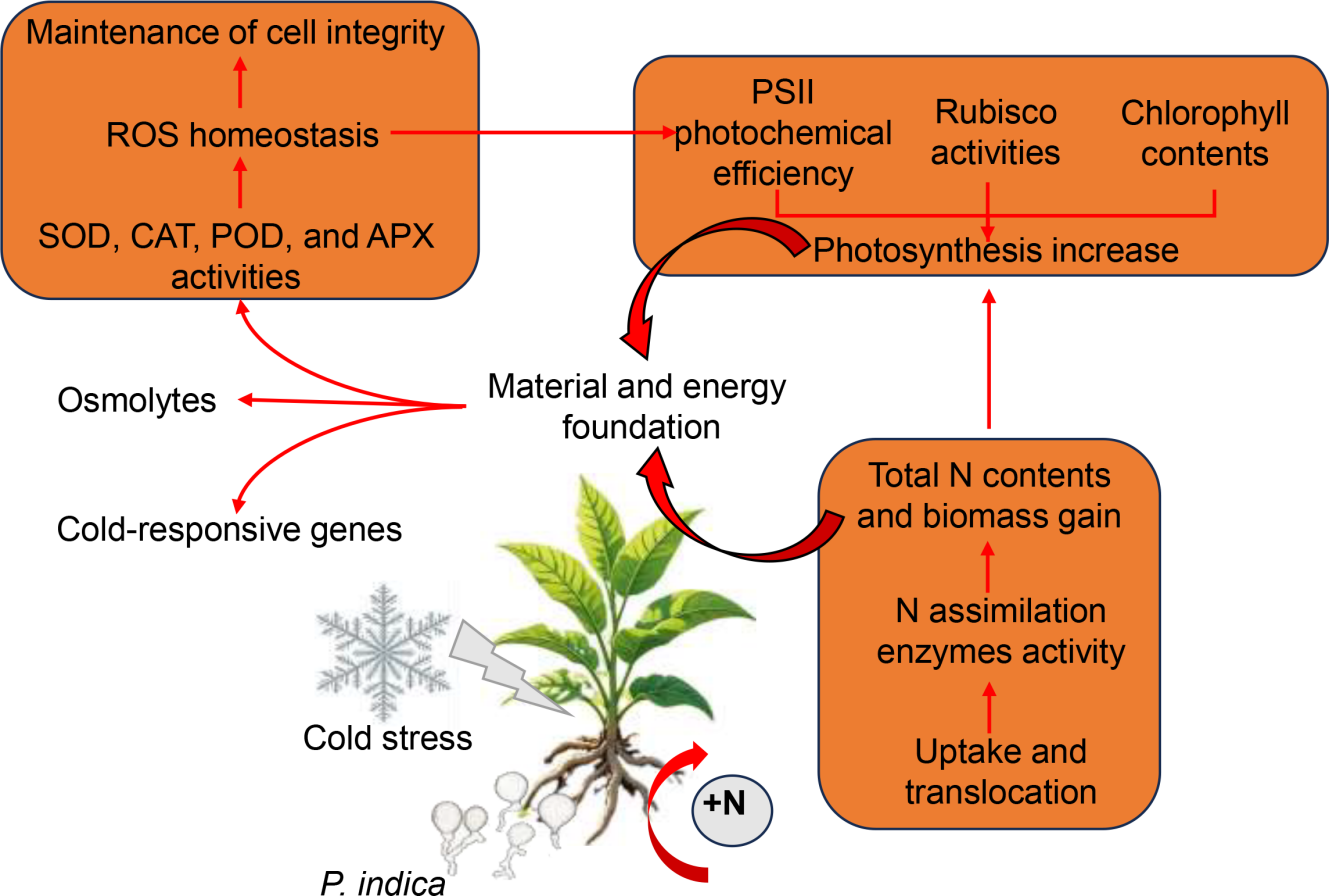
Proposed model of enhanced cold stress tolerance in tobacco through root colonization by *Piriformospora indica*. The orange boxes indicate strongly induced processes, and the red arrows represent positive regulatory processes.

In summary, under low-temperature stress, *P. indica* enhances the activity of antioxidant enzymes in host plants, thereby facilitating the elimination of ROS and mitigating both peroxide-induced damage to plant cells and the detrimental effects of excessive ROS generation on the PSII reaction center (**Figure 9**). Additionally, *P. indica* stimulates the accumulation of osmolytes, contributing to the restoration of osmotic balance (**Figure 9**). When exposed to cold stress, *P. indica* not only promotes photosynthesis in tobacco by stimulating the synthesis of photosynthetic pigments, enhancing Rubisco activity, and improving PSII efficiency, but also strengthens tobacco’s nitrogen assimilation by inducing the expression of nitrate transporter genes and activating enzymes related to nitrogen assimilation (**Figure 9**). The resultant improvement in nitrogen use efficiency further promotes photosynthetic activity by enhancing the allocation of nitrogen to photosynthetic enzymes, pigment content, and light absorption. This synergistic optimization of nitrogen and carbon assimilation provides a solid material and energetic foundation for tobacco plants to withstand cold stress (**Figure 9**). Furthermore, *P. indica* confers cold resistance to tobacco by stimulating the expression of cold-responsive genes (**Figure 9**). Our findings provide a valuable insight into the mechanisms by which *P. indica* enhances cold tolerance in tobacco and underscore the potential of using symbiotic fungi to improve crop resilience to abiotic stresses.

## Data Availability

The original contributions presented in the study are included in the article/**Supplementary Material**. Further inquiries can be directed to the corresponding authors.
