# Supplementary material for: Enhancing cold tolerance in tobacco through endophytic symbiosis with Piriformospora indica
Source: Front Plant Sci. 2024 Oct 25;15:1459882. doi: 10.3389/fpls.2024.1459882 (PMC11543411; doi:10.3389/fpls.2024.1459882)
Supplement: Supplementary file 1 [file DataSheet1.pdf]

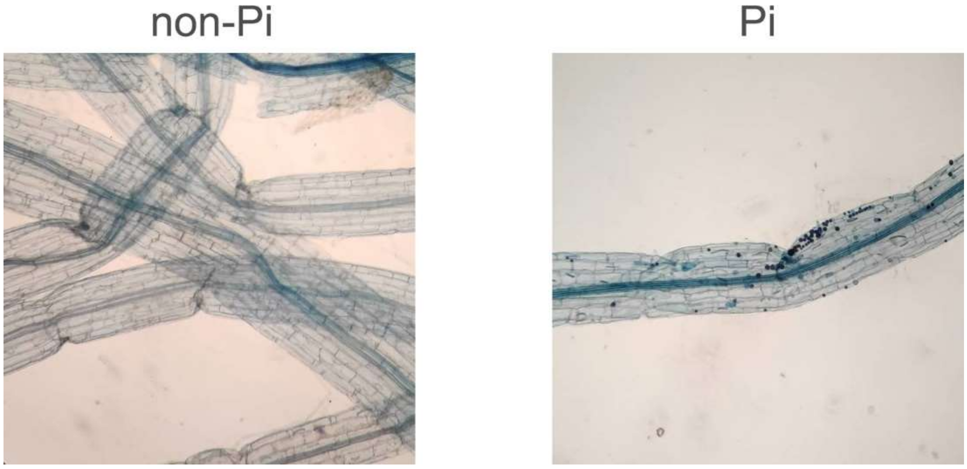

Figure S1. *Piriformospora indica* colonization in tobacco root. non-Pi: roots of non-inoculated control; Pi: Roots of *P. indica*-colonized tobacco.
